# *Tetrahymena* Metallothioneins Fall into Two Discrete Subfamilies

**DOI:** 10.1371/journal.pone.0000291

**Published:** 2007-03-14

**Authors:** Silvia Díaz, Francisco Amaro, Daniel Rico, Virginia Campos, Laura Benítez, Ana Martín-González, Eileen P. Hamilton, Eduardo Orias, Juan C. Gutiérrez

**Affiliations:** 1 Departamento de Microbiología-III, Facultad de Biología, Universidad Complutense (UCM), Spain; 2 Department of Molecular, Cellular, and Developmental Biology, University of California Santa Barbara, Santa Barbara, California, United States of America; Wellcome Trust Sanger Institute, United Kingdom

## Abstract

**Background:**

Metallothioneins are ubiquitous small, cysteine-rich, multifunctional proteins which can bind heavy metals.

**Methodology/Principal Findings:**

We report the results of phylogenetic and gene expression analyses that include two new Tetrahymena thermophila metallothionein genes (MTT3 and MTT5). Sequence alignments of all known Tetrahymena metallothioneins have allowed us to rationalize the structure of these proteins. We now formally subdivide the known metallothioneins from the ciliate genus Tetrahymena into two well defined subfamilies, 7a and 7b, based on phylogenetic analysis, on the pattern of clustering of Cys residues, and on the pattern of inducibility by the heavy metals Cd and Cu. Sequence alignment also reveals a remarkably regular, conserved and hierarchical modular structure of all five subfamily 7a MTs, which include MTT3 and MTT5. The former has three modules, while the latter has only two. Induction levels of the three T. thermophila genes were determined using quantitative real time RT-PCR. Various stressors (including heavy metals) brought about dramatically different fold-inductions for each gene; MTT5 showed the highest fold-induction. Conserved DNA motifs with potential regulatory significance were identified, in an unbiased way, upstream of the start codons of subfamily 7a MTs. EST evidence for alternative splicing in the 3′ UTR of the MTT5 mRNA with potential regulatory activity is reported.

**Conclusion/Significance:**

The small number and remarkably regular structure of Tetrahymena MTs, coupled with the experimental tractability of this model organism for studies of in vivo function, make it an attractive system for the experimental dissection of the roles, structure/function relationships, regulation of gene expression, and adaptive evolution of these proteins, as well as for the development of biotechnological applications for the environmental monitoring of toxic substances.

## Introduction

Metallothioneins (MTs) constitute a superfamily of ubiquitous low molecular weight (<7–10 kDa), cysteine-rich proteins that bind heavy metal ions (mainly Cd, Zn and Cu) via metal-thiolate clusters [1]. Typically these proteins have 18 to 23 highly conserved cysteine residues and lack aromatic amino acids and histidine. MTs are found, not only throughout the animal kingdom, but also in other eukaryotes (protists and higher plants) and in prokaryotic microorganisms [2].

MTs are multifunctional proteins that are involved in many diverse biological processes. Their primary biological role remains obscure; they perform a range of functions depending on the specific needs of the particular organism or tissue and its environmental circumstance. Some of the known functions of MTs [1] include: essential-metal homeostasis [3], protection against heavy metal toxicity by sequestration [4], trapping reactive oxygen species (ROS) [5], and protection against xenobiotics [6]. In mammals, they have also been implicated in protection against neurodegenerative diseases [7] and in development and cellular differentiation processes [8]. While not essential proteins [9]–[11], MTs do seem to provide organisms with a survival advantage during times of stress.

*Tetrahymena thermophila* and *T. pyriformis* are excellent eukaryotic unicellular model organisms for the study of toxic compounds in the environment [12]–[15]. Their physiology, biochemistry and molecular biology are well characterized; pure cultures grow quickly and inexpensively in completely defined or complex nutrient media; and they are readily portable to field conditions. Advanced genetic technology available in *T. thermophila* [16], will permit experimental analysis of *in vivo* MT gene function.

The first ciliate cadmium-binding MTs (CdMT) were isolated from *Tetrahymena pyriformis* and *Tetrahymena pigmentosa* [17] and their primary amino acid sequence determined. The corresponding gene of *Tetrahymena pyriformis* (*MT-1*, abbreviated *TpMT-1* in this article) was sequenced in 1999 [18]. Additional *Tetrahymena* MT sequences have since been reported: the *MTT1*. *MTT2*, and *MTT4* genes of *T. thermophila* [19]–[20], the *MT-2* gene of *T. pyriformis* ([21]; abbreviated here as *TpMT2*) and the *MT-2* gene of *T. pigmentosa* ([22]; abbreviated here as *TpigMT2*).

*Tetrahymena* MTs have generally been considered to fall into two groups, which show relatively little sequence identity with one another. One group is most efficiently induced by cadmium (*TpMT-1* and *MTT1*), while the other responds best to copper (*TpigMT2*, and *MTT2* and *MTT4* from *T. thermophila*) ([19], [21]–[23] and W. Miao, pers. comm.). The mRNA and protein levels of *TpMT-1* and *MTT1* increase significantly with Cd concentration in the medium [19], [23], [24]. Other agents, such as Hg, Cu, hydrogen peroxide, heat, and interleukin-6 also induce MTT1, but to a lesser extent [23]. Thus MTT1 is not strictly a heavy metal-responsive protein, but is induced under more general stress conditions.

Genes encoding two new *T. thermophila* metallothionein isoforms (*MTT3* and *MTT5*) have been identified; their characterization is reported in this article. Sequence comparisons have revealed a regular, conserved and hierarchical modular structure that the predicted proteins, MTT3 and MTT5, share with MTT1, TpMT-1 and TpMT2. The level of expression of each gene in response to a variety of stressors was determined using quantitative RT-PCR. Like *MTT1*, *MTT3* and *MTT5* are more efficiently induced by Cd than Cu, and behave as multi-stress response proteins. Potential regulatory motifs have been computationally identified in the flanking sequence of *MTT1, MTT3* and *MTT5*. Alternative splicing, which occurs in the 3′ UTR of *MTT5*, can potentially affect its expression. A phylogenetic analysis and consideration of distinguishing structural features have led us to formally subdivide the *Tetrahymena* MTs into two subfamilies that correlate well with their differential inductive responses to Cd and Cu.

## Results

### Structural characterization of two new MT genes in Tetrahymena thermophila

To isolate new metallothionein genes in T. thermophila we used PCR and degenerate primers based on the sequence of the MT-1 gene of *T. pyriformis*, MET1 and MET2 (see Table S1). A portion of a new MT gene, *MTT3* was isolated as a 380 bp product amplified from whole-cell genomic DNA (from SB210 and BI3840), as was a ∼600 bp product which matched the *MTT1* gene, which has been independently isolated from various strains of this ciliate [19], [23].

**Figure 1:**
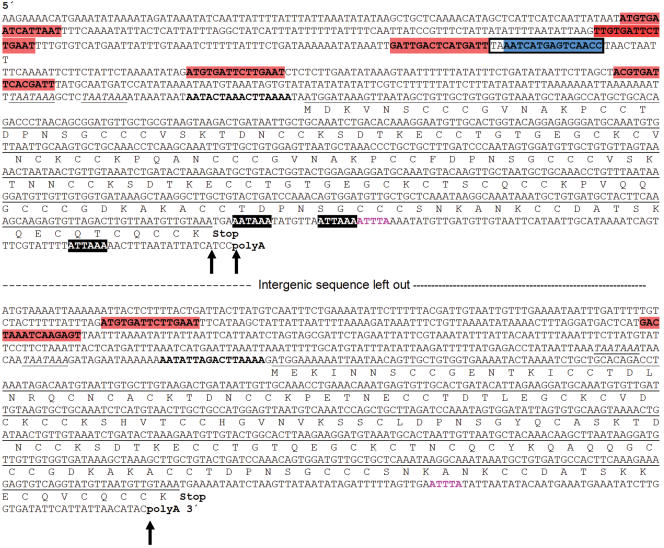
Nucleotide sequences of the *MTT1* and *MTT3* genes and flanking regions. Selected segments of the complete macronuclear sequence assembly (about 4.4 Kb) including two *Tetrahymena thermophila* MT genes (*MTT1* and *MTT3*). Amino acid sequences (one-letter code) are shown beneath the underlined coding regions of *MTT1* and *MTT3*. Various motifs in sequence flanking both coding regions are indicated on the nucleotide sequence: MTCM1 (Metallothionein Conserved Motif 1) plus and minus strand matches (red and blue highlighted sequences, respectively); a perfect palindrome (boxed region) formed mostly by two inverted MTCM1 motif copies; a 16 nt conserved sequence in the putative 5′UTR of both genes (bold letters); TATA-like boxes (underlined italic letters); potential polyadenylation signals (white letters in black boxes); and potential mRNA degradation signal (bold pink letters in 3′ flanking region). The poly-A tail sites in *MTT1* (DQ517937 and EF195745) and *MTT3* (EF195744) are indicated by arrows.

The complete *MTT3* coding sequence was originally obtained using PCR products from a genomic library of 5–7 Kb inserts (see Materials and Methods). DNA sequences from five overlapping clones generated an assembly of 4,418 bp. Its nucleotide sequence (partially shown in Fig. 1) revealed that the *MTT1* and *MTT3* genes lie adjacent to each other 1.7 Kb apart, with the same orientation.

Once the whole-genome sequence became publicly available (http://www.tigr.org/tdb/e2k1/ttg/), the entire ∼4.4 kb sequence containing *MTT3* and *MTT1* was found to perfectly match a single macronuclear (MAC) chromosome scaffold (8254373; 875 kb) which was identified as the physical counterpart [25] of genetic coassortment group ML04R, and mapped to the left arm of micronuclear (MIC) chromosome 4 (see Supplemental Table 4 in [26]). This MAC chromosome has now been completely sequenced (see assembly 4985 in the “Closedscaffolds.fasta” folder at ftp://ftp.tigr.org/pub/data/Eukaryotic_Projects/t_thermophila/Assemblies_and_Sequences/Closed_new/). A diligent search of the entire genome and predicted proteome for sequences related to *MTT1* and *MTT3* revealed that they are each single-copy genes and detected the existence of only one other putative MT gene (*MTT5*; see below).

An open reading frame with in-frame triplets coding for start (AUG) and stop (UGA) sites for *MTT3* was identified by alignment to the *MTT1* sequence. The predicted translation start fits the statistical properties of start codons in *T. thermophila* [27]. A cDNA clone isolated from a Cd-treated gene expression library (EF195744) confirmed that this gene is expressed, lacks introns in the coding sequence, and identified a poly A addition site (Figure 1). Several *MTT3* ESTs are also present in Genbank (CX576384, CX591981, CX574735). *MTT3* encodes a putative protein of 162 amino acids which has 76% amino acid identity to the MTT1 protein. In fact, these genes are so similar we were unable to distinguish their expression levels by Northern blot analysis due to the strong cross-hybridization of the *MTT1* and *MTT3* probes (data not shown).

The second new putative MT gene (*MTT5*) was found by BLAST searching the *T. thermophila* genome or predicted gene sequences (at the TIGR website: http://www.tigr.org/tdb/e2k1/ttg/) for sequences similar to *MTT1* or its predicted protein. This gene and its expression have been validated; its cDNA sequence (DQ517936) was obtained from several clones in gene expression libraries (from Cd or As treated cell populations). Each of our cDNA sequences aligns very well with the *MTT5* cDNA sequence submitted to GenBank by Santovito et al. (AY884209; data not shown). *MTT5* resides on scaffold 8254577 (349,481 bp closed), which maps to micronuclear chromosome 5 and corresponds to genetic coassortment group BD34R (see Supplemental Table 4 in [26]).

**Figure 2:**
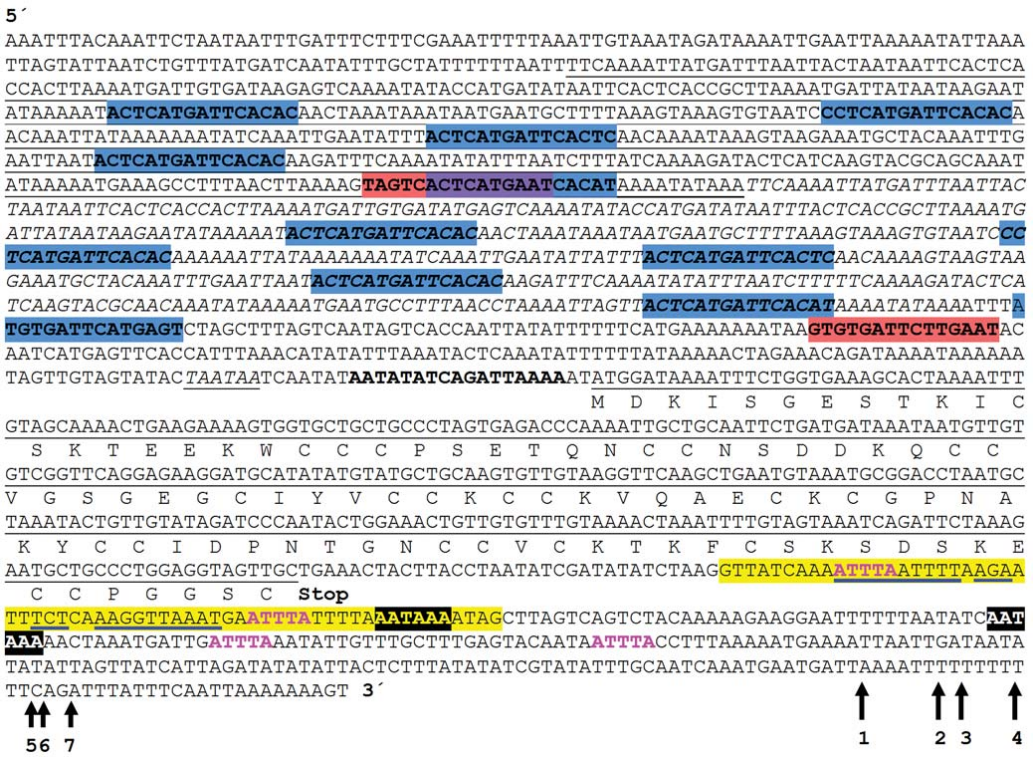
Nucleotide sequence of the *MTT5* gene and flanking regions. Motifs shared with *MTT1* and *MTT3* are indicated as in Figure 1. Additional features: a segment of partial red and blue overlap between two inverted copies of MTCM1 is shaded lavender; a 476-bp duplicated region (one duplicate is underlined, the other italicized); a 63 bp intron in the 3′UTR is highlighted yellow, with blue underscoring to indicate a 32-nucleotide potential stem-and-loop structure; Numbered arrows: PolyA addition sites inferred, from GenBank cDNA and EST submissions, as the junction between genomic sequence and a run of at least 5 As absent from it. The physical addition site is ambiguous when the genomic sequence contains one or more adenines at the right of the junction, as defined. (Accession numbers corresponding to each arrow: 1: CX576461, CX576877 and DQ517936; 2: CX577509; 3: CX576383; 4: CX572435; 5: CX573352 and CX578296; 6: CX581879, CX590118, and our unpublished observations; 7: CX574235.

The 297 bp coding region of *MTT5* contains an open reading frame that encodes a predicted protein of 99 amino acids (Fig. 2). Alignment of our cDNA sequence to the genomic sequence revealed the existence of a 63 bp intron, beginning at position +30 downstream of the TGA stop codon, thus well outside the protein-coding sequence (Fig. 2). The intron has canonical donor and acceptor splice sites and represents the first intron to be described in a *Tetrahymena* MT transcript. Inspection of the cDNA submitted to GenBank by Santovito et al. and 17 ESTs downloaded by us from NCBI also confirm this intron. Surprisingly, four independent ESTs in the NCBI EST database (CX573730, CX576461, DY683553, and CX579679) retain the intron; they align perfectly with the *MTT5* genomic DNA. The presence of both the spliced and unspliced versions of the *MTT5* RNA was further validated by RT-PCR (Figure S1). We consider this strong evidence for alternative splicing of the *MTT5* transcript – to our knowledge the first case of alternative splicing reported in *T. thermophila*.

**Figure 3:**
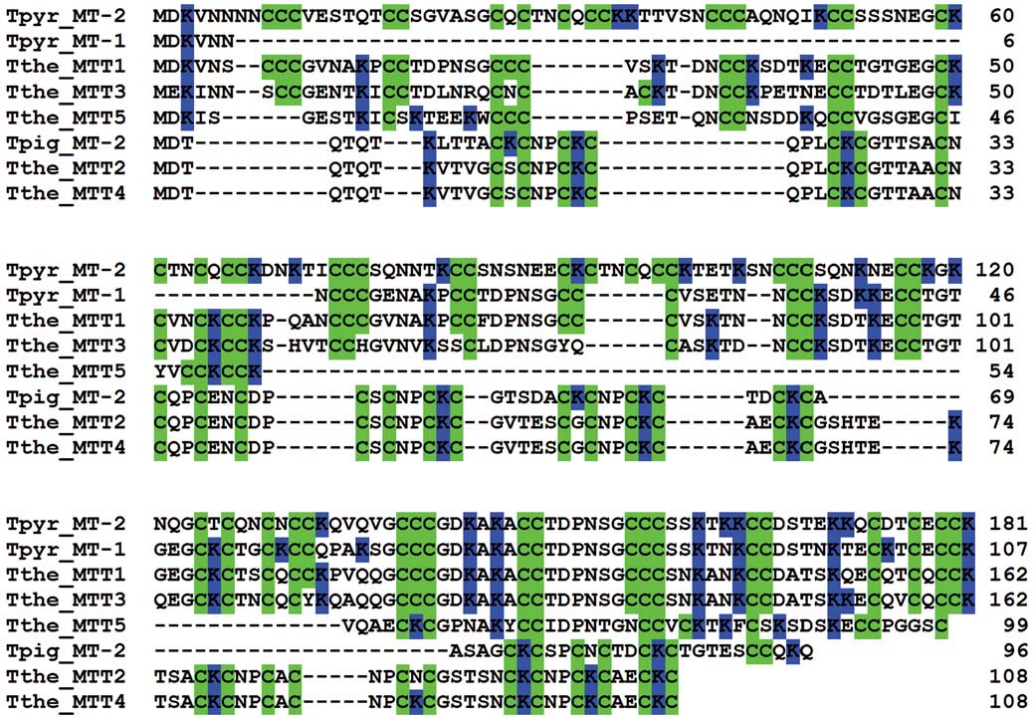
Alignment of eight *Tetrahymena* metallothioneins. Key to *Tetrahymena* species abbreviations: Tthe = *T. thermophila*, Tpyr = *T. pyriformis*; Tpig = *T. pigmentosa*. The protein sequences were aligned using the T-Coffee software with default settings without any manual adjustments. Cys and Lys residues are highlighted in green and blue, respectively. Numbers represent the position of the last amino acid in each row. The top 5 proteins correspond to subfamily 1, while the bottom three belong in subfamily 2, as defined in the text.

Two alternative polyA addition sites were also identified (Fig. 2). At least sites for polyA addition in *MTT5* can be inferred from *T. thermophila* ESTs available in GenBank (CX573730, CX576461, DY683553, CX579679, CX577509, DY683476, CX590118, CX578271, CX577821, CX574235, CX573352, CX572581, CX583643, CX581560, CX576383, CX578296, CX576877, CX572734, CX577823, CX581879, CX572435; data not shown).

### Interesting structural features of the MTT3 and MTT5 proteins

Several structural features of MTT3 and/or MTT5 deserve special notice:

- Exceptional MT length. The MTT3, and MTT1 predicted proteins are exceptionally long (162 amino acids each) in comparison to the usual range of 24–75 amino acids for MTs of other organisms (1). Indeed, their lengths fall in the top 1% of the >500 non-redundant Cys-rich (>25% Cys) proteins in GenBank that are annotated as metallothionein(-like).
- CCC and CC clusters. Both proteins have a high number of Cys residues, even for cysteine-rich MTs. As was also noted for the first described ciliate MT (TpMT-1, [17]), MTT3 and MTT5 are unusual in having clusters of 3 consecutive cysteines. As will be described below, this distinctive feature is shared with MTT1 and TpMT2.
- Only a minority of Lys and Ser residues occur next to Cys residues, a feature shared with MTT1, TpMT-1 and TpMT2. This stands in contrast to mammalian MTs, where it was noticed early on that Lys and Ser residues have a strong tendency to be juxtaposed with Cys residues [28]–[31].
- Modular structure. Finally, MTT3 and MTT5, along with certain other *Tetrahymena* MTs, possess an unmistakable modular structure, described in more detail in the next section.

### Sequence alignments of *Tetrahymena* metallothioneins reveal distinct subfamilies and a hierarchical modular structure

A multiple alignment of MTT3 and MTT5 proteins with all other described and confirmed *Tetrahymena* MTs was carried out using T-coffee software with default settings. The alignments (Fig. 3) show that the MTs fall into two groups, readily distinguished by the type of cysteine residue clustering. In the first group (upper 5 lines in the alignment), the majority of cysteines are arranged in clusters of two or three consecutive residues. In contrast, consecutive Cys residues are virtually absent in the second group.

A second, relatively independent structural feature also distinguishes the two groups, namely the physical relationship of Lys to Cys residues along the polypeptide backbone. The Lys residues of the MTs in the smaller group have a strong tendency to be juxtaposed with Cys (4.7 to 1 juxta- to non-juxtaposed ratio), as in mammalian MTs. In contrast, in the MTs in the larger group, including MTT1, MTT3 and MTT5, Cys-juxtaposed Lys residues are the minority (0.7 to 1 ratio). The locations of Lys residues have been highlighted in Fig. 3.

**Figure 4:**
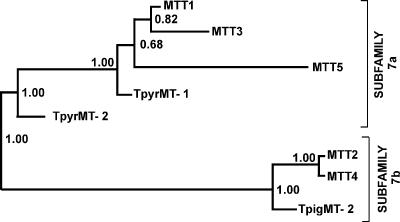
Phylogenetic tree of 8 *Tetrahymena* MTs. The alignments shown in Fig 3 were used to construct the tree using Mr. Bayes (mixed protein model). The tree was rooted at its midpoint. The numbers represent the posterior probabilities that each branching is correct.

A phylogenetic tree was constructed by submitting the alignment in Fig. 3 to the “Mr. Bayes” software ([32], using a “mixed protein” model). The tree (Fig. 4) reinforces the distinctions between the two groups identified above: the posterior probability that the two branches are correctly separated is 1.0. Ciliate MTs have been assigned to MT family 7 [33]. Based on this phylogenetic analysis, on the organization of cysteine clusters, and on the types of elementary building blocks assembled during the evolution of these proteins (see below), we define two subfamilies of *Tetrahymena* MTs. Subfamily 7a consists of *T. thermophila* MTT1, MTT3 and MTT5 and *T. pyriformis* MT-1 and MT-2. Subfamily 7b consists of *T. thermophila* MTT2 and MTT4, as well as *T. pigmentosa* MT-2.

**Figure 5:**
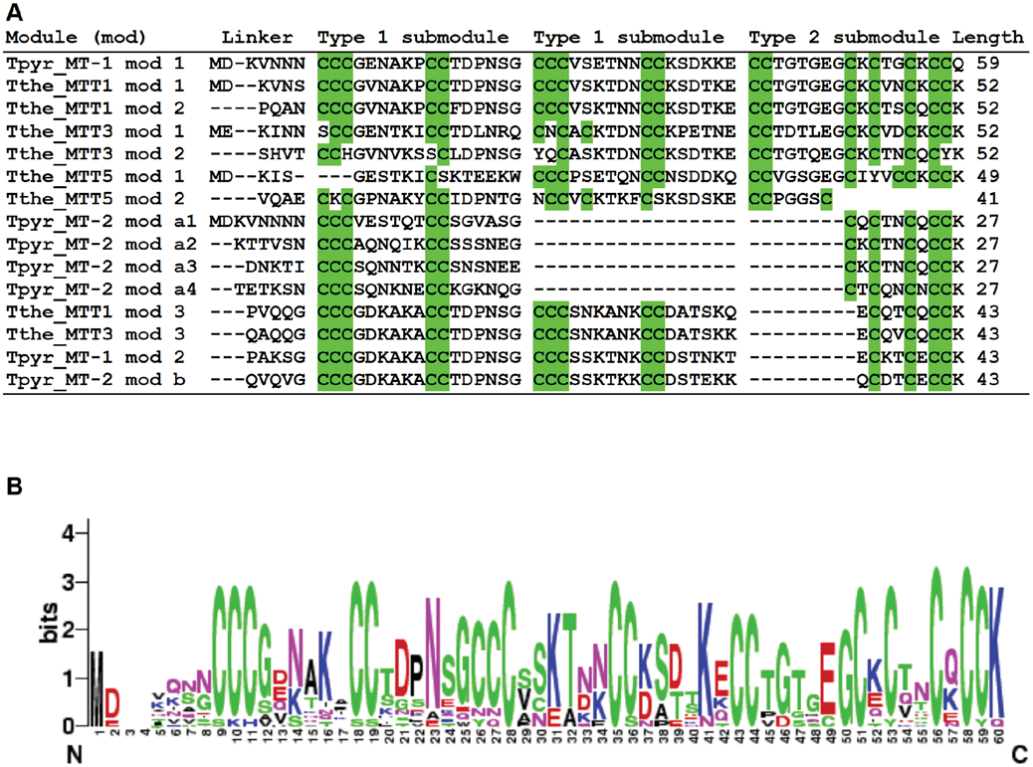
Members of *Tetrahymena* subfamily 7a metallothioneins are composed of modules. Panel A. Alignment of all 15 modules. The MTs were divided up into modules as described in the text; multiple alignments were made using the T-coffee program, followed by visual inspection and manual adjustment. Linkers and submodules (see text) have been separated from one another by a blank column. The bottom four lines represent the highly conserved C-terminal modules of four MTs, missing in MTT5. Panel B. Amino acid conservation at every module position. The height of each letter represents the extent of conservation. At any given position in the module alignment, diversity is minimal (and conservation and logo height maximal) when every module has the same amino acid and there is no gap in any module. Conversely, a blank (0-height) logo column represents minimal conservation, i.e., maximal diversity, at that module position.

All five members of *Tetrahymena* MT subfamily 7a are readily and unambiguously divided up into segments initially defined by the criterion that every segment carries the CXCCK motif at its C-terminus; we call these segments “modules”. They vary in length from 27 to 59 amino acids and are separated by 4–6 bp “linkers”. Multiple alignments of all these modules were made using the T-coffee program, followed by visual examination and manual adjustment, and are shown in Fig. 5.

Closer inspection of the alignments reveals several interesting features:

- First, cysteines have highly conserved positions within modules, so Cys residues are readily aligned “in register” (Fig. 5). TpMT-1 is missing precisely one module-equivalent, independently supporting the proposed modular organization and the criteria used to delimit modules.
- Second, the entire 48-amino acid C-terminal modules of the MTT3, MTT1, TpMT-1 and TpMT2 proteins are remarkably conserved: all four modules are identical to one another at 34 out of 44 positions, including a continuous 22-amino acid segment (i.e., the entire first type 1 submodule, defined below), and have conservative substitutions at all but one of the remaining positions.
- Third, the modules appear to result from the compounding of two types of submodules (1 and 2, shown in Fig. 5). Type 1 submodules have the sequence C_3_X_6_C_2_X_6_, while the complete type 2 submodules can be represented as C2X6CXCXXCXCCK. Four TpMT2 modules are missing one copy of a type 1 submodule, further supporting the proposed submodular structure. Interestingly, no sequences resembling type 1 submodules are seen in the 7b subfamily.
- Fourth, the structure of these submodules suggests that they were built by combining two elementary building blocks, the 8-amino acid C_2_X_6_ motif and the 10-amino acid CXCXXCXCCK motif. The latter motif is found exclusively at the C-terminus of every module and is absent only in module 2 of MTT5. Two conserved, abbreviated forms of submodule 2 are seen, one in the modules from the N-terminal two thirds of TpMT2 and a distinct one shared by the C-terminal modules of the MTT3, MTT1, TpMT-1 and TpMT2 proteins. The Cys-juxtaposed Lys residues are concentrated in submodule 2. A slightly truncated version of the second building block (CKCXXCKC) has been used extensively in the evolutionary construction of the *Tetrahymena* subfamily 7b MTs (see Fig. 3).

### Expression analysis of *MTT1*, *MTT3*, and *MTT5* by real-time quantitative PCR

**Figure 6:**
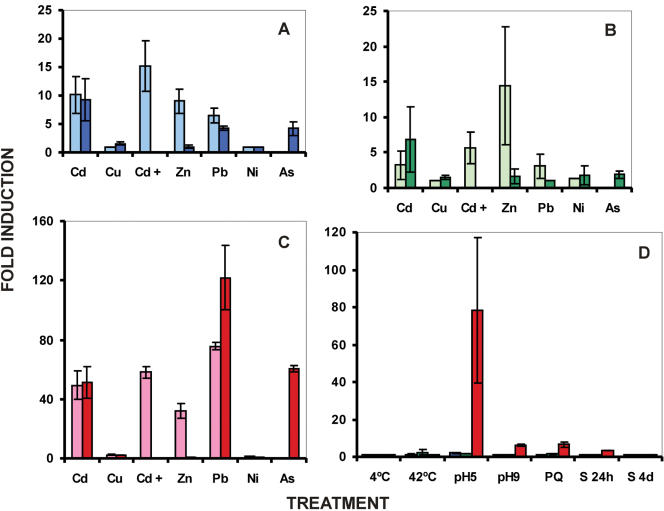
Relative expression levels of *Tetrahymena thermophila* *MTT1*, *MTT3* and *MTT5* genes obtained by quantitative RT-PCR. Panels A, B and C: fold-induction for each gene (*MTT1*, *MTT3*, and *MTT5*, respectively) after treatment by different heavy metals. Gene expression levels are shown relative to an untreated control (which is set at 1±0.0 for every gene). Normalization of expression was achieved against the amplification of an endogenous gene (α-tubulin). Each bar of the histogram corresponds to an average value ±SD of two or three independent experiments. Heavy metal concentrations are as reported in the Material & Methods; Cd+ refers to 27 µM Cd plus 80 µM Cu; treatments times were: 1 h (light bars) and 24 h (dark bars) for all heavy metals. See Table S2 for numerical values. Panel D. Relative expression levels for each gene, in the order *MTT1* (blue bars), *MTT3* (green bars) and *MTT5* (red bars), after induction by stress treatments other than heavy metals. Temperature treatments were for 2h, pH and Paraquat treatments were for 24 h, while starvation treatments were for 24 hrs and 4 days.

Comparative gene expression analysis of the three *T. thermophila* subfamily 7a genes was carried out under various stress conditions using real-time quantitative RT-PCR. In Fig. 6 and Table S2 we show the relative mRNA expression levels of *MTT1*, *MTT3* and *MTT5* induced by various stressors, normalized against the level of α-tubulin (the reference gene). *MTT5* consistently showed the highest induction levels, relative to α-tubulin, while *MTT3* showed the weakest induction. Heavy metal treatments result in generally similar induction patterns for the three genes. Cd is a strong inducer of every gene, but Pb seems to preferentially induce *MTT5*. None of the MT genes showed significant induction by Ni. Regarding other types of stressors, *MTT5* was strongly and preferentially induced by acid pH. It was also the only MT gene significantly induced by basic pH (pH 9) or oxidative stress (PQ) and was weakly induced by starvation (24 h treatment). Although there was some variability in our measurements, *MTT5, like MTT1,* clearly behaves as a multi-stress response protein, with *MTT5* responding to the widest range of stressors.

### Search for conserved motifs in the regions flanking the *MTT1*, *MTT3* and *MTT5* genes

Conserved transcription-related motifs, shared by the *T. thermophila MTT1*, *MTT3* and *MTT5* genes, were searched for in their 5′- and 3′- flanking regions (see Materials and Methods). Using two multiple-alignment programs, MEME and the Gibbs sampler, we found a robust motif, here named the Metallothionein Conserved Motif 1 (MTCM1). The best possible match is GTG*TGA*A*TC*A*TGA*G*T*, where highly conserved nucleotide positions (frequency at least 90%) are underscored. Matches are shown in Fig. 1 (*MTT1* and *MTT3*), Fig. 2 (*MTT5*) and Table S3. This motif (MTCM1) is present upstream of *MTT1* (six times), *MTT3* (two times) and *MTT5* (thirteen times) and it also occurs upstream of *TpMT-1* (five times). Although the copies of the motif belong to the same sequence family, hints of gene-specific differentiation can be detected between *MTT5* and the *MTT1-MTT3* pair (see Table S3).

A whole-genome sequence search, using the 21 sequences of the MTCM1 copies found upstream of the three MTT genes, yielded only four additional occurrences of this motif, none of which occurred within predicted exons. Three are upstream of predicted genes whose function is not well characterized (see Table S3), and one is within an intron near the 3′ end of a long coding sequence. Those four MTCM1 occurrences were at locations in the genome unrelated to those of any MTT genes or one another, and thus showed none of the clustering which characteristically occurs upstream of the three MTT genes of family 7a (Fig. 1 and 2).

**Figure 7:**
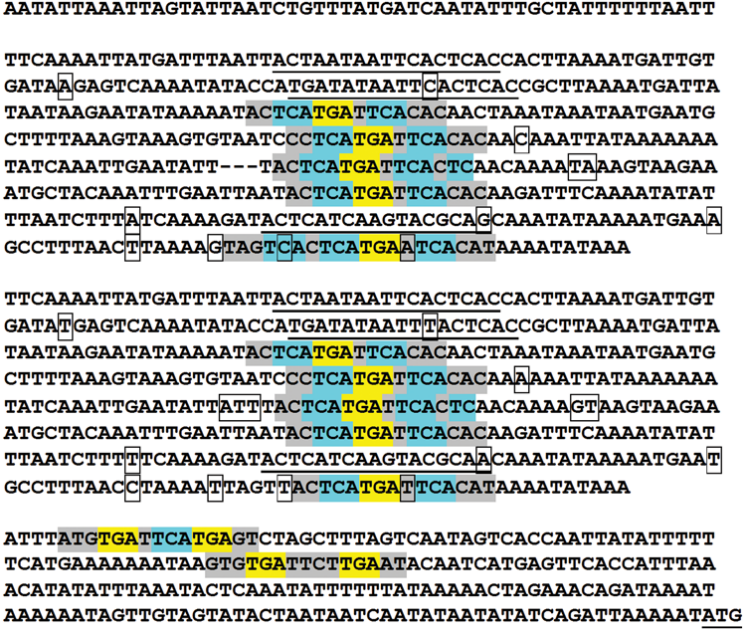
Hierarchical modular structure of the DNA sequence in the *MTT5* 5′ flanking region. The two major tandem 416-bp repeats are delimited by blank lines; a three-bp gap (dashed) has been introduced in the first repeat in order to facilitate visual detection of the two repeats. Grey highlight: MTCM1 motif; underlined: degraded versions of MTCM1. Aqua (TCA) and yellow (TGA) shading within MTCM1: elements of the AP1 binding site-like heptanucleotide TGA(A/T)TCA, and of a TCATGA palindromic hexanucleotide that overlaps the AP1-like site; this shading is included to facilitate visual identification of the orientation of various MTCM1 copies. Nucleotide substitutions between the two repeats are boxed. Underlined ATG at the end of the sequence: *MTT5* start codon.

The 5′-UTR of *MTT5* has an unusual characteristic; a 416 bp tandem duplication in the putative promoter region (Figs. 2 and 7). The first copy is located from −212 to −628; the second copy (from −629 to −1,042) is contiguous with the first, but 3 bp shorter (Fig. 2). The copies are 96% identical to one another. The five copies of MTCM1 motif within each repeat show a striking periodicity of approximately every 54 bp (Fig. 7). The MTCM1 motif is the most conserved segment within the 54-bp repeat units.

BLAST alignments detected a 16-bp conserved sequence within 2–3 bp of the translation start sites of *MTT1, MTT3* and *MTT5* (Figs. 1 and 2). Percent identities range between 70 and 87%. The conservation of their sequence and location relative to the start codon is suggestive of regulatory significance for the expression of these genes.

Additional eukaryotic regulatory motifs have been highlighted in Figs. 1 and 2: TATA box (TAATAAA), polyadenylation signals (AATAAA and ATTAAA) and mRNA degradation signal (ATTTA). Because these motifs are embedded in the very AT-rich intergenic sequence characteristic of *Tetrahymena* and the functions of these motifs have not been experimentally confirmed for any gene in this organism, and thus remain hypothetical, we will not discuss them further.

## Discussion

### Characteristics of two new metallothioneins from Tetrahymena thermophila

The Tetrahymena subfamily 7a proteins (MTT3 and MTT5 included) are unusually large in comparison to the usual size range of 24–75 amino acids for MTs of other organisms [1]. There is no evidence of introns in the coding sequence of any known Tetrahymena MT gene; in this respect they resemble those of cyanobacteria [2], and stand in contrast to those of other eukaryotes [1], [34].

MTs are rich in cysteines, which participate in metal binding [35], [36]. The Tetrahymena subfamily 7a MTs have more cysteine residues (up to 54 residues) than most MTs (generally only 18–23 cysteine residues [35]). The cysteines (C) are arranged in clusters of 2 and 3 consecutive residues that are very well conserved among the five members of the Tetrahymena MT subfamily 7a. In contrast, CCC clusters are completely missing and CC clusters are very rare in subfamily 7b MTs. The CCC motif is infrequently found in the MTs of other organisms, rare examples are: a CdMT from the annelid Eisenia fetida (P81695), an MT from the yeast Yarrowia (Candida) lipolytica (P41928) and a CuMT from the arthropod Callinectes sapidus (AAF08966). However, the whole-genome sequencing of an increasing number of organisms has revealed small, cysteine-rich “hypothetical” proteins that contain CCC clusters (our unpublished observations), which are potentially good candidates for novel MTs.

Chemical considerations early suggested a role for CXC clusters as an initial nucleation site in the final multi-cysteine coordination of heavy metal in mammalian MTs [28]. Convergent evolution guided by the requirements of metal complexation is thought to be responsible for the occurrence of Cys-X-Cys and Cys-Cys sequences in otherwise unrelated MTs [37]. Interestingly, such motifs occur exclusively near the C-terminal submodules of subfamily 7a MTs, while they are distributed throughout the sequence of subfamily 7b MTs. The striking differences in the clustering and spacing of cysteine resides in the *Tetrahymena* MT subfamilies, are likely to provide useful material for the experimental identification of structure-function differences between MTs of the two subfamilies.

A general feature of MTs is that they tend to lack aromatic amino acids and histidine [1], [35]. MTT1 most stringently obeys this rule with only one phenylalanine residue, while MTT3 and MTT5 are less typical. There is some precedent for such atypical MTs, for example, MTs from cyanobacteria and several plant Type 1 MTs [38]–[39]. A striking feature of the Tetrahymena subfamily 7a MTs is the extreme asymmetry in the ratio of the positively charged amino acids, lysine and arginine. In total, the five MTs contain 83 lysine residues but only one arginine residue (in MTT3). We have likewise observed a strong asymmetry in the K/R ratio in mammalian MTs (not shown). When coupled with the observed underrepresentation of the aliphatic side-chain amino acids, Leu and Ile, the above observations may simply reflect a more general phenomenon, i.e. an underrepresentation of amino acids with bulky side chains in MTs. Perhaps a strong hydrophobic internal core would impose structural constraints which would be deleterious to at least one function of the protein.

### Our analyses define two metallothionein subfamilies in the ciliate genus Tetrahymena

Ciliate MTs were placed in a family of their own, family 7 ([33], http://www.expasy.ch/cgi-bin/lists?metallo.txt), because of the distant relationship of TpMT-1, the only ciliate MT described at the time, to other MTs. In the meantime, the sequences of seven additional *Tetrahymena* MTs have become available, including the two described here. Based on four criteria – our phylogenetic analysis of the eight *Tetrahymena* MTs, the organization and spacing of their cysteine clusters, the location of Lys relative to Cys residues, and the differential induction of gene expression by the heavy metals Cd and Cu – we now formally subdivide family 7 into two subfamilies (Figs. 3 and 4). The diagnostic characteristics of each subfamily are summarized in Table 1. Subfamily 7a includes *T. thermophila* MTT1, MTT3 and MTT5 and *T. pyriformis* MT-1 and MT-2. Subfamily 7b consists of *T. thermophila* MTT2 and MTT4, as well as *T. pigmentosa* MT-2. The Bayesian phylogenetic analysis yields a 1.0 posterior probability that these two groups are correctly separated (Fig. 4). The difference in the fraction of juxtaposed Lys and Cys residues is large enough to allow every one of the eight *Tetrahymena* MTs to be assigned to the correct group with 99% certainty based on this criterion alone. No additional MT subfamilies are detected in the fully sequenced *T. thermophila* genome. A MEME computational search for peptide motifs in the eight *Tetrahymena* MTs yielded motifs shared by subfamily 7a or 7b proteins, but none shared by both.

**Table 1:** Diagnostic characteristics of MT subfamilies 7a and 7b

MT subfamily	Cys residues in CC or CCC clusters	Elementary sequence motifs	Lys next to Cys residues	Fold induction by Cd and Cu**
7a	Most	C_2_X_6_,most common; CKCXXCXCCK*	Minority	Cd>Cu
7b	Essentially none	CKCXXCKC	Strong majority	Cd<Cu

C, K and X = cysteine, lysine and any amino acid other than C, respectively.

* Consensus sequence. In subfamily 7a this motif is present exclusively at C-termini of type 2 submodules, which in turn exclusively constitute the C-termini of modules.

** To the extent reported so far: *MTT1* [20]; this work); *MTT3* and *MTT5* (this work); *TpigMT-1* and *TpigMT-2* [19]; *TpMT1* and *TpMT2* [18]; *MTT2* and *MTT4* (L-F Feng and W. Miao, personal communication).

MT isoforms in many other organisms exhibit functional differentiation to favor binding by either divalent metals (Cd and Zn) or monovalent copper [40]–[44]. MTT1 and TpMT-1 are induced by and efficiently bind Cd [17], [21], [23], while TpigMT2, MTT2, and MTT4 are induced by and bind Cu ([20], [45] and W. Miao personal communication). Based on these properties, we tentatively identify subfamily 7a as cadmium (and zinc) thioneins and subfamily 7b as copper thioneins.

### Hierarchical modular organization of the *Tetrahymena* subfamily 7a MTs

Alignment of the protein sequences of the subfamily 7a MTs (Fig. 5) reveals a remarkably regular and hierarchical modular organization of these proteins. They appear to have evolved through the combinatorial accretion of repeat units of increasing length and complexity, built from two elementary types of motifs, C_2_X_6_ and CKCXXCKCCK (consensus). With only minor modifications, these motifs have been successively combined into submodules, modules and finally MTs (Fig. 5). The size of the modules, their separation by “linkers” consisting of 4–6 amino acids, and the conservation of differences between modules across the proteins suggests a likely functional significance of the modules, which may well represent independently folded domains.

The modular structure of the subfamily 7a MTs could have contributed to their unusually large size by facilitating the evolutionary expansion of functional capacity during episodes of gradual environmental pollution. The potential speed of such an internal repeat-dependent evolutionary process has been recently demonstrated for a surface protein of the yeast *S. cerevisiae* [46].

The precise evolutionary history of subfamily 7a proteins may be difficult to unscramble. Embedded in the modular structure of these proteins is evidence of some mixing and matching of submodule sequences. Two factors, active during the evolution of these proteins, may have contributed to this confusion. First, the hierarchical modular structure may have facilitated successive expansion and contraction of the number of gene subunits coupled with possible recombinational events between paralogs, such as unequal crossing-over or gene conversion. Second, since MTs are multifunctional proteins, their structures may have been subjected to a constant evolutionary “push and pull” by competing functional pressures which could have resulted in some convergent evolution of particular structural subunits.

### Stressor effects on gene expression of *T. thermophila* subfamily 7a MTs

The quantitative real-time RT-PCR analysis of the expression of the three genes (*MTT1*, *MTT3* and *MTT5*) shows that *MTT5* is the most strongly induced (i.e. shows the highest fold-induction) and *MTT3* the least induced by diverse environmental stressors (Cd, Pb, Cd+Cu, As, and acid pH). A duplicated DNA segment in the *MTT5* putative promoter region, which includes several presumed recognition sites for an AP-1-like transcription factor (Fig. 7), may be related to the high level of induction of this gene when compared to the *MTT1* and *MTT3* genes. Although all three MTs are strongly induced by Cd, at least MTT1 and MTT5 are multi-stress response proteins because they are induced by a range of environmental stressors (Figure 6, Table S2).

*MTT1* and *MTT3* encode very similar MT isoforms (76% identity) and undoubtedly originated by gene duplication; subsequent changes in their promoter regions may be responsible for their differential response. Divergence of expression is proposed to be the first step in the functional divergence of duplicate genes, and to increase the likelihood that both genes will be retained in the genome [47]–[49]. Furthermore, for duplicate genes, expression differences are positively correlated with divergence of *cis*-regulatory motif(s) [50], although changes in other factors, such as mRNA stability and chromatin structure, can also be responsible for expression differences. The paucity of putative regulatory motifs (MTCM1) in the *MTT3* promoter may contribute to the somewhat lower induction levels observed for this gene relative to *MTT1*.

Dondero et al. [23] examined the induction of *MTT1* by various stressors using real-time quantitative PCR. Despite observing different fold-inductions, both studies agree that Cd is the best inducer of *MTT1*. Disparities in the relative expression levels measured by the two studies may be due to differences in experimental conditions (e.g. culture medium, stressor concentration, length of exposure) and/or in the quantitative RT-PCR protocol. In our hands, a one hour Zn treatment significantly induced *MTT1*. Dondero's group observed some induction of the *MTT1* gene by Cu, but none by Zn. This was somewhat surprising as Zn and Cd have been shown to be potent inducers of MT transcription and protein synthesis in other organisms [1], [35], [51]. Dondero et al. [23] used a substantially lower concentration of Zn (4 to 100 µM versus our 870 µM, which we selected using the metal's LC_50_ value for this ciliate); the lower concentration may explain why they failed to see induction. In addition, it has been shown that quantitative RT-PCR can give significant variation and non-reproducibility between different laboratories, even with identical samples [52].

Interestingly, Pb treatment induces the expression of all three MT genes; indeed *MTT5* achieved its highest expression level in the presence of Pb (Figure 6C). Metallothionein or metallothionein-like proteins have been shown to be induced by lead in rats [53], humans [54] and fish [55]. Furthermore, Pb is second to Cd in its ability to displace Zn from hepatic Zn-metallothionein [56] and it can displace Cd from the CdMT complex [57]. In plants [58], transcriptome analysis reveals that many genes respond similarly to Pb and Cd (half of the genes that changed their expression in Pb-treated plants also changed their expression in Cd-treated ones). Pb bioaccumulation has also been related to an over-expression of MTs and phytochelatin synthases [59]

The high level of expression of *MTT5* induced by acid pH is potentially valuable from a biotechnological standpoint. *T. thermophila* is a very favorable microbial eukaryote for the high level expression of foreign proteins [60]–[61]. The advantages of the *MTT1* and *MTT2* 5′ flanking regions for gene over-expression have already been highlighted [19]–[20]. Promoters of genes that respond strongly to non-toxic inducers (such as *MTT5* in cells exposed to pH 5) are potentially useful for the industrial production of proteins intended for internal use in the treatment of human disease.

### A comparative analysis of potential transcription-related elements among Tetrahymena CdMTs

MT genes of many organisms are regulated at the level of transcription. The promoter regions of the mammalian MT-1 and MT-2 genes contain multiple metal responsive elements (MREs) and glucocorticoid responsive elements (GREs), as well as elements involved in basal transcription. A metal responsive, zinc finger transcription factor (MTF-1) binds to promoter proximal MREs to induce transcription (see review [51]). Similar MREs are present in the promoters of Drosophila MT genes; a homolog of the mammalian MTF-1 protein activates their transcription in response to heavy metals [62]. While the T. thermophila genome contains numerous Zn finger proteins, none appears to be a true ortholog of MTF-1 and no canonical MRE motifs were identified by our MEME computational search. But this is not surprising. Even in S. cerevisiae, which phylogenetically is more closely related than Tetrahymena to metazoa, the trans-acting regulatory protein (ACE1), which binds to tandem upstream activating sequences in the presence of copper and silver ions to activate transcription of a CuMT gene (CUP1) [63], is unrelated to MTF-1.

Very few *cis*-acting regulatory elements have been identified experimentally in *Tetrahymena* [64], and those few are generally in connection with special genes. A conserved motif (MTCM1) was identified in the 5′-flanking regions of *T. thermophila MTT1*, *MTT3* and *MTT5* genes and the *T. pyriformis* homolog, *TpMT-1,* by using a computational method free of preconceptions. The conserved motif MTCM1 is present in all subfamily 7a MTs included in the search; six times in *MTT1,* with two inverted copies contributing sequence to a perfect palindrome (Fig. 1), five times in *TpMT-1*, two times in *MTT3* and 13 times in *MTT5* (Fig. 2)). Several points suggest that this motif may well have functional significance relatively specific to the family 7a MT genes: a) Clusters of multiple, highly statistically significant matches are found within 500 bp upstream of the start codon of the four tested subfamily 7a MTs. b) MTCM1 sequence conservation spans two related species (*T. thermophila* and *T. pyriformis*). c) No detectable sequence conservation surrounds the conserved motif in each gene. d) The motif is detected at only four additional locations in the genome, and those do not spatially cluster with one another (Table S3). The tight specificity of this putative regulatory motif implies the existence of a transacting factor(s) that is relatively specific for MT genes. This factor would confer the ability to manipulate the expression of the MTT genes in coordinate fashion, and perhaps as a more selectively targeted component of a general stress response.

Most of the MTCM1 copies include a sequence (TGANTCA, where “N” means any nucleotide) related the sequence of the element that binds the eukaryotic AP-1 transcription factor (TGAG/CTCA) [65]. Thus a transcription factor with AP1-like binding specificity may have a regulatory function in the expression of the subfamily 7a MTs. In *Saccharomyces cerevisiae* [66], an AP-1 transcription factor (YAP-1) is involved in the response to oxidative stress and metal resistance; indeed deletion mutants in the gene encoding this transcription factor are hypersensitive to the presence of cadmium in the medium.

Although there are hints of periodicity in the location of the MTCM1 motif copies in *MTT1* and *TpMT-1* (see Table S3), nowhere is periodicity as striking as in the *MTT5* 5′ flanking region. This periodicity is strongly suggestive of earlier, extensive tandem duplications of a 54-bp segment. The motif copies are significantly more conserved than the surrounding sequence, which suggests that they play an important functional role. There is less sequence conservation of 53-bp repeat units within 415-bp repeat units than between repeat units at corresponding locations along the 415-bp duplicate copies (Figure 7). This strongly suggests that all the episodes of 53-bp subunit duplication and most of their diversification pre-date the tandem 415-bp duplication.

### EST evidence for alternative splicing of the *MTT5* mRNA: potential regulatory significance

The intron located in the 3′ UTR of *MTT5* may play a role in regulating MT expression. The existence of seemly authentic *MTT5* mRNAs with and without this intron supports the first case of alternative intron splicing reported in *T. thermophila*. The *MTT5* intron does not involve the coding sequence; thus, any functional significance of the alternative splicing should be regulatory. In Ciliates only one other case has been reported, in *Euplotes raikovi*, where alternative splicing determines whether a mating pheromone remains anchored to the cell surface and functions as a receptor or is released as a soluble protein to the surrounding medium, where it functions as a chemoattractant and as an inducer of mating-reactivity [67]–[68].

Determining the role of the alternative splicing lies outside the scope of the present report, but it is interesting to note that the intron removes two putative mRNA degradation motifs, but leaves two others intact (Fig. 2). In addition, sequences within the intron have the potential to form a 32-nucleotide stem and loop structure (see blue underlined regions in Figure 2) and could significantly alter the secondary structure of the 3′ UTR. Secondary structure and CACC repeats in the 3′ UTR of the rat *MT1* mRNA are thought to be responsible for its localization to the perinuclear cytoplasm [69]. Messenger RNA localization is a mechanism which allows eukaryotic cells to synthesize proteins close to where they function. This targeting of newly synthesized mRNA usually involves localization signals (or zipcodes) within the 3′ UTR of the transcript (see reviews [70]–[71]).

## Materials and Methods

### Ciliates and culture conditions

Two Tetrahymena thermophila strains with inbred strain B genetic background were used; BI3840 [72] and SB210 [25]. All Tetrahymena strains were grown axenically in PP210 medium (2% w/v aqueous solution of proteose peptone (Difco), supplemented with 10 µM FeCl_3_ and 250 µg/ml each of streptomycin sulphate and penicillin G, all three from Sigma), and maintained at a constant temperature of 32±1°C. Ultrapure reagent grade H_2_O was used in all experiments, with maximum conductivity of 18.2 MΩ, obtained using a MILLI-Q water purification system (Millipore).

### Stress treatments

Prior to RNA isolation, Tetrahymena thermophila (SB210) cultures were exposed to different stress conditions. LC_50_ values were determined for each heavy metal. Concentrations used were approximately half the LC_50_ value and resulted in negligible cell mortality. Cells were treated for 1 or 24 hours with each of the following heavy metals: 27 µM Cd (CdCl_2_, Sigma), 80 µM Cu (CuSO_4_. 5H_2_O, Sigma), 870 µM Zn (ZnSO_4_. 7H_2_O, Sigma), 604 µM Pb (Pb(NO_3_)_2_, Sigma), 421 µM Ni (NiCl_2_. 6H_2_O, Sigma), or 24 µM As (NaHAsO_4_. 7H_2_O, Sigma). Temperature stress was a 2-hr cold (4°C) or heat (42°C) shock, while pH stress took place during 24 hrs at high (pH 9) or low (pH 5) pH. Oxidative stress was induced by treating the cells for 24 hrs with 778 µM Paraquat (Sigma). All the above treatments were in PP210 medium. Starvation was in Dryl's buffer (1.7 mM sodium citrate, 2.4 mM sodium phosphate, 2 mM CaCl_2_, pH 7.4) for 24 hrs or 4 days.

### Total DNA and RNA isolations

Exponential cultures (100 ml) of Tetrahymena were harvested by centrifugation at 700×g for 2 min. Total DNA was isolated as described [73]. Total RNA was isolated using the RNAqueos^TM^- 4PCR kit (Ambion). All samples were treated with RNase-free DNase I (Ambion), according to the protocol supplied by the manufacturer.

Total RNA was isolated from conjugating cells using the RNeasy mini kit (Qiagen), according to the manufacturer's directions for animal cells. Tetrahymena strains of differing mating types (SB210 and SB 1969) were starved in 10 mM Tris, pH 7.5 and mated as described [74]. RNA was isolated from conjugating cells at 3, 5, and 10 hours after conjugation was started by mixing separately starved cultures. All samples were subjected to on-column DNase digestion according to the manufacture's directions.

### Standard PCR reactions and cloning

Convergent degenerate primers, MET1 and MET2 (Table S1), were designed using the amino acid sequence of MT-1 [17]. 50 µl PCR reactions contained: 1× PCR buffer (GeneAmp Gold buffer), 2 mM MgCl_2_, 0.2 mM each dNTP, 0.2 mM each primer, 1.25 units/µl AmpliTaq Gold DNA polymerase (PE Applied Biosystems) and 5 µl of DNA (∼100 ng). Template DNA was either genomic DNA or cDNA. Amplification was: 7 min at 94°C, 30 cycles of 1 min at 94°C, 1 min at 50°C and 2 min at 72°C; and 72°C for 5 min. PCR products were analyzed by standard 1.5% agarose gel electrophoresis in TAE buffer (40 mM Tris, 1 mM EDTA and 5.7% glacial acetic acid) stained with ethidium bromide (1 µg/ml). The DNA size standard was “Marker VI” (0.15–2.17 kb) from Roche. Amplified PCR products were cloned, using the TOPO TA Cloning kit (Invitrogen).

### Library screening by long range PCR

A macronuclear (MAC) library of 5–7 kb random inserts from T. thermophila (SB210) in the pCC1FOS vector (Epicentre) was screened for MTT3 flanking sequences. Convergent (LC and RC) and divergent (LD and RD) primers, designed using the partial MTT3 gene sequence, and vector primers (VL2 and VR3) are shown in Table S1. The library was screened with all eight pairwise combinations of one specific primer with one vector primer, using long-range PCR amplifications with KOD polymerase (Novagen), according to the manufacturer's directions. PCR products were cloned using the TOPO TA Cloning kit (Invitrogen).

### RT-PCR

RT-PCR for intron analysis was carried out using the StrataScript One-Tube RT-PCR System (Stratagene) according to the manufacture's directions. All RNA samples were treated with DNase I (amplification grade; Invitrogen) prior to amplification. Table S1 shows the primers used to confirm alternative splicing of the MTT5 intron. MTT5L and MTT5R flank the intron while MTT5IL and MTT5 IR span the intron junctions (see Fig. S1).

### Quantitative real-time RT-PCR

The cDNA synthesis was carried out using 5 µg RNA, oligo d(T) primer (5 µM), AMV reverse transcriptase (Roche) (75 U/µg RNA) with RNase inhibitor (25 U) (Roche) and 2.5 mM dNTPs, in a total volume of 20 µl. cDNA samples were amplified in duplicate in 96 microtiter plates (Applied Biosystems). Each PCR reaction (20 µl total volume) contained: 10 µl of SYBR Green PCR Master Mix (Applied Biosystems), 1 µl of each primer (at 300 nM final concentration), 3 µl of H_2_O and 5 µl of cDNA. Quantitative PCR primers for each MT gene (MTT1 A & B, MTT3 A & B, MTT5 A & B) and the α-tubulin gene (ATUB 1 & 2), which was used as an endogenous control gene or constitutive cDNA, are showed in Table S1. The size and sequence of each PCR product (54 bp for α-tubulin, 192 bp for MTT1, 156 bp for MTT3 and 79 bp for MTT5) was confirmed by gel electrophoresis and DNA sequencing.

Real-time PCR reactions were carried out in an ABI PRISM 7700 real time PCR apparatus, and the thermal cycling protocol was as follows: 10 min at 95°C, followed by 40 cycles (15 seconds at 95°C and 1 min at 50°C). All controls were negative (no template control and RT minus control). The specificity of each primer pair was tested by qPCR; a unique PCR product was obtained for all primer pairs as determined by melting curve analysis.

To calculate the relative change in expression we used the ΔΔCt method [75]. Quantification was done relative to the reference gene (α-tubulin) by subtracting the cycle threshold (C_T_) of the reference gene from the C_T_ of the gene of interest; the resulting difference in cycle number is “ΔC_T_”. ΔC_T_s obtained under control conditions (i.e. no stress) were subtracted from those obtained under a particular stress treatment, to give the “ΔΔC_T_”. The fold induction is 2^-ΔΔCt^. This method requires that the PCR amplification efficiencies of all genes be similar and preferably at or above 90%. Amplification efficiency, the fraction of template that gets replicated per PCR cycle, was measured by using 10-fold serial dilutions of a positive control PCR template and plotting C_T_ as a function of log [13] concentration of template. The slope of the resulting trend line is a linear function of the PCR efficiency. A slope of - 3.32 indicates 100% amplification efficiency. The efficiency requirement was met for all the genes tested; their amplification efficiencies were greater than 99% and the correlation coefficients (R^2^) were all 99% (Table S4).

### DNA sequencing and sequence analysis

The DNA sequences were determined using an ABI PRISM^TM^ 377 DNA automatic sequencer (PE Applied Biosystems) or an ABI 310 Genetic Analyzer (inserts from MAC library screening) according to the dideoxy technique, using appropriate primers and synthetic oligonucleotides (Big-dye^TM^ terminator cycle sequencing ready reaction kit from AP Biosystems). Inserts from at least two independent clones were sequenced, off both strands. Homology searches were performed using BLAST program at the NCBI website (http://www.ncbi.nlm.nih.gov/BLAST/). Multiple sequence alignments were carried out with Align X using the software program Vector NTI^TM^ Suite 9 (InforMax) and the GCG Wisconsin Package (Accelerys). Additional primers were designed to completely sequence large cloned fragments.

### Searching for transcription-related motifs and phylogenetic analysis

To detect conserved motifs related to regulation of gene expression lying upstream or downstream of the MT genes, the multiple sequence alignment programs MEME and Gibbs Sampler were used [76]–[77], as well as alignment in pairwise combinations using the BLAST 2 sequences webpage at NCBI. To search for degenerate motifs identified in other organisms, the “DNA pattern find” program of The Sequence Manipulation Suite 2 website (http://bioinformatics.org/sms2/) was used. Preliminary *T. thermophila* sequence data was obtained from The Institute for Genomic Research website at http://www.tigr.org.

### Multiple sequence alignments and phylogenetic tree construction

Protein sequences were aligned using the T-coffee (Tree-based Consistency Objective Function For alignmEnt Evaluation, [78]) server (http://www.ch.embnet.org/software/TCoffee.html), under default settings. Amino acid conservation at every position of the peptide sequence alignment was plotted using the “sequence logo” method [79], by pasting the alignment at the logo website (http://weblogo.berkeley.edu/logo.cgi, [80]). At a given position, amino acid conservation is inversely related to amino acid diversity, which in turn is measured using the classical, base-2 logarithmic definition of information content in “bits” [81].

A phylogenetic tree of the *Tetrahymena* MTs was constructed starting with the T-coffee alignment and using MrBayes software under a “mixed protein” model [82]. The Markov Chain Monte Carlo (MCMC) search was run until the standard deviation of split frequencies fell below 0.01.

### Nucleotide sequence accession numbers

The MTT3 and MTT5 (cDNA) sequences have been deposited in the GenBank Database: AY740525 and EF195744 (MTT3) and DQ517936 (MTT5).. GenBank accession numbers of other Tetrahymena MT genes referred to in this article are: T. pyriformis MT-1 (TpMT-1), AJ005080; MT-2 (TpMT2), AY765220; T. pigmentosa MT-1, AF509328; MT-2, AF479586; T. thermophila MT-1, AF479587; MTT1, AY061892, AF537326, AY273793, DQ517937, and EF195745; MTT2, AY204351; MTT4, AY660008.

## Supporting Information

Table S1 PCR primers. All sequences are written 5′ to 3′. Primers were designed using the software at the Primer 3 website (http://frodo.wi.mit.edu/cgi-bin/primer3/primer3_www.cgi, [83]). Primers for quantitative RT-PCR were designed using the software Primer Express v2.0 (Applied Biosystems). Degenerate primers (MET1 and MET2): R = A or G; W = A or T; Y = C or T.
(0.04 MB DOC)

Table S2 Relative expression levels of Tetrahymena thermophila MTT1, MTT3 and MTT5 genes obtained by quantitative RT-PCR. Gene expression levels are shown relative to an untreated control (which is set at 1±0.0 for every gene). Normalization of expression was achieved against the amplification of an endogenous gene (α-tubulin). Two or three independent experiments were used to calculate the average values ±SD for each gene. Bold numbers are significantly different from control at p<0.01. Heavy metal concentrations and other stress treatments were as reported in the Material & Methods
(0.08 MB DOC)

Table S3 MTCM1 motif copies found in the entire T. thermophila genome and upstream of the TpMT-1 gene: Sequences, match locations, and nearest gene. Matches: listed in the same orientation; TCA and TGA trinucleotides have been highlighted in aqua and yellow to facilitate motif sequence comparisons and identification of the AP-1-binding-related element (TGANTCA). Nearest genes: named MT genes or predicted gene model identifiers; sequence, coordinates, functional annotation and EST support for gene models are available by searching the Tetrahymena Genome Database (http://www.ciliate.org/). For the T. pyriformis MT-1 gene (TpMT-1), the GenBank accession number and coordinates relative to the translation start site are listed. Note: G nucleotides at position 14 have been shaded in grey to illustrate a hint of systematic gene-specific differentiation of the motif that can be detected between MTT5 and the MTT1-MTT3 T. thermophila gene pair.
(0.07 MB DOC)

Table S4 Quantitative real-time RT-PCR standard curve parameters for each MT gene and the expression control
(0.03 MB DOC)

Figure S1 Alternative Splicing of the MTT5 3′UTR intron. A. Location of primers used for RT-PCR relative to the intron in the 3′ UTR of MTT5. The intron is shown in red, while the flanking regions are blue. The RT-PCR products produced by each set of primers (arrows) and their expected sizes are shown below. Primer sequences are shown in Table S1. B. RT PCR analysis using RNA extracted from conjugating Tetrahymena cells. Lanes 1 and 8: 1 kb DNA ladder (Invitrogen). Lanes 2 and 3: PCR products produced by primers flanking the intron (MTT5L and MTT5R). The predicted 158 bp product (templated by spliced mRNA) and a fainter 221 bp product (templated by unspliced RNA) are RT-dependent, i.e., seen only in the “+RT” (plus reverse transcriptase) lane. Lanes 4 and 5: one primer spans the left boundary of the intron (MTT5IL) and the other flanks it on the right (MTT5R). The PCR product is RT-dependent and has the size (173 bp) expected if it is templated by unspliced RNA. Lanes 6 and 7: the left flanking primer (MTT5L) in combination with a primer which spans the right hand junction of the intron (MTT5IR). The PCR product is RT-dependent and has the size (145 bp) expected if it is templated by unspliced RNA.
(0.35 MB TIF)
