# Supplementary material for: Tetrahymena Metallothioneins Fall into Two Discrete Subfamilies
Source: PLoS One. 2007 Mar 14;2(3):e291. doi: 10.1371/journal.pone.0000291 (PMC1808422; doi:10.1371/journal.pone.0000291)
Supplement: Table S1 — PCR primers. All sequences are written 5′ to 3′. Primers were designed using the software at the Primer 3 website (http://frodo.wi.mit.edu/cgi-bin/primer3/primer3_www.cgi, [83]). Primers for quantitative RT-PCR were designed using the software Primer Express v2.0 (Applied Biosystems). Degenerate primers (MET1 and MET2): R = A or G; W = A or T; Y = C or T. (0.04 MB DOC) [file pone.0000291.s001.doc]

**Table S1. PCR primers**

| MET1 | AAYTGYTGYTGYGGWGARAAY |
| --- | --- |
| MET2 | TCRGTRCARCARGCYTTRGCY |
| VL2 | GTGCTGCAAGGCGATTAAGTTGG |
| VR3 | CTCGTATGTTGTGTGGAATTGTGAGC |
| LC | ATGCAAATGTGTTGATTGTAAGTGCT |
| RC | CATCCTTCTTAAGTGCCAGTACAACA |
| LD | AGCACTTACAATCAACACATTTGCAT |
| RD | TGTTGTACTGGCACTTAAGAAGGATG |
| ATUB1 | TGTCGTCCCCAAGGAT |
| ATUB2 | GTTCTCTTGGTCTTGATGGT |
| MTT1A | TGTAAATGCTAAGCCA |
| MTT1B | AAAGCAGCAGGGTTTAG |
| MTT3A | ACAATGTAATTCTCTT |
| MTT3B | TAAGCAGCTGGATTTGA |
| MTT5A | TGTGTCGGTTCAGGAGAAGGA |
| MTT5B | AGGTCCGCATTTACATTCAGCTT |
| MTT5L | CGATATATCTAAGGTTATCAAAATTTAATTTTAAG |
| MTT5R | TCTTTTTGTAGACTGACTAAGCTATTTTATTT |
| MTT5IL | TAAAGAATGCTGCCCTGGAG |
| MTT5IR | AAAGGTAAATTATTGTACTCAAAGCAAAC |

All sequences are written 5’ to 3’. Primers were designed using the software at the Primer 3 website (<http://frodo.wi.mit.edu/cgi-bin/primer3/primer3_www.cgi>, [83]). Primers for quantitative RT-PCR were designed using the software Primer Express v2.0 (Applied Biosystems). Degenerate primers (MET1 and MET2): R = A or G; W = A or T; Y = C or T.
