# Supplementary material for: Tetrahymena Metallothioneins Fall into Two Discrete Subfamilies
Source: PLoS One. 2007 Mar 14;2(3):e291. doi: 10.1371/journal.pone.0000291 (PMC1808422; doi:10.1371/journal.pone.0000291)
Supplement: Table S2 — Relative expression levels of Tetrahymena thermophila MTT1, MTT3 and MTT5 genes obtained by quantitative RT-PCR. Gene expression levels are shown relative to an untreated control (which is set at 1±0.0 for every gene). Normalization of expression was achieved against the amplification of an endogenous gene (α-tubulin). Two or three independent experiments were used to calculate the average values ±SD for each gene. Bold numbers are significantly different from control at p<0.01. Heavy metal concentrations and other stress treatments were as reported in the Material & Methods (0.08 MB DOC) [file pone.0000291.s002.doc]

**Table S2**  **Relative expression levels of *Tetrahymena thermophila* *MTT1*, *MTT3* and *MTT5* genes obtained by quantitative RT-PCR**

| Treatment | Exposure  Time (hr) | MTT1 | MTT3 | MTT5 |
| --- | --- | --- | --- | --- |
| Cd | 1 | **10.1**$\pm$ 3.3 | **3.26** $\pm$ 2 | **49.38** $\pm$ 9.5 |
| Cd | 24 | **9.32** $\pm$ 3.7 | **6.79** $\pm$ 4.6 | **51.17** $\pm$ 10.9 |
| Cu | 1 | 1 $\pm$ 0 | 1 $\pm$ 0 | 2.41 $\pm$ 0.3 |
| Cu | 24 | 1.53 $\pm$ 0.3 | 1.44 $\pm$ 0.3 | 2.22 $\pm$ 0.04 |
| Cd + Cu | 1 | **15.2** $\pm$ 4.5 | **5.65** $\pm$ 2.3 | **58.04** $\pm$ 4.1 |
| Cd + Cu | 24 | 0 $\pm$ 0 | 0 $\pm$ 0 | 0 $\pm$ 0 |
| Zn | 1 | **9** $\pm$ 2.2 | **14.4** $\pm$ 8.3 | **32.1** $\pm$ 5 |
| Zn | 24 | 1 $\pm$ 0.3 | 1.62 $\pm$ 1 | 1 $\pm$ 0 |
| Pb | 1 | **6.5** $\pm$ 1.3 | **3.1** $\pm$ 1.7 | **75.7** $\pm$ 2.4 |
| Pb | 24 | **4.3** $\pm$ 0.4 | 1 $\pm$ 0 | **121.8** $\pm$ 21.6 |
| Ni | 1 | 1 $\pm$ 0 | 1.3 $\pm$ 0 | 1.2 $\pm$ 0.2 |
| Ni | 24 | 1 $\pm$ 0 | 1.76 $\pm$ 1.3 | 1 $\pm$ 0 |
| As | 1 | 0 $\pm$ 0 | 0 $\pm$ 0 | 0 $\pm$ 0 |
| As | 24 | **4.23** $\pm$ 1.2 | 1.9 $\pm$ 0.5 | **60.4** $\pm$ 1.9 |
| 4^o^C | 2 | 1 $\pm$ 0 | 1 $\pm$ 0 | 1 $\pm$ 0 |
| 42^o^C | 2 | 1.33 $\pm$ 0.3 | 2.5 $\pm$ 1.3 | 1 $\pm$ 0 |
| pH 5 | 24 | 2 $\pm$ 0.1 | 1.64 $\pm$ 0 | **78.3** $\pm$ 38.7 |
| pH 9 | 24 | 1 $\pm$ 0 | 1 $\pm$ 0 | **6.13** $\pm$ 0.6 |
| Paraquat | 24 | 1 $\pm$ 0 | 1.47 $\pm$ 0.2 | **6.63** $\pm$ 1.5 |
| Starvation | 24 | 1 $\pm$ 0 | 1 $\pm$ 0 | **3.32** $\pm$ 0.17 |
| Starvation | 96 | 1 $\pm$ 0 | 1 $\pm$ 0 | 1 $\pm$ 0 |

Gene expression levels are shown relative to an untreated control (which is set at 1 ± 0.0 for every gene). Normalization of expression was achieved against the amplification of an endogenous gene (α-tubulin). Two or three independent experiments were used to calculate the average values ± SD for each gene. Bold numbers are significantly different from control at p < 0.01. Heavy metal concentrations and other stress treatments were as reported in the Material & Methods
