# Supplementary material for: Tetrahymena Metallothioneins Fall into Two Discrete Subfamilies
Source: PLoS One. 2007 Mar 14;2(3):e291. doi: 10.1371/journal.pone.0000291 (PMC1808422; doi:10.1371/journal.pone.0000291)
Supplement: Table S3 — MTCM1 motif copies found in the entire T. thermophila genome and upstream of the TpMT-1 gene: Sequences, match locations, and nearest gene. Matches: listed in the same orientation; TCA and TGA trinucleotides have been highlighted in aqua and yellow to facilitate motif sequence comparisons and identification of the AP-1-binding-related element (TGANTCA). Nearest genes: named MT genes or predicted gene model identifiers; sequence, coordinates, functional annotation and EST support for gene models are available by searching the Tetrahymena Genome Database (http://www.ciliate.org/). For the T. pyriformis MT-1 gene (TpMT-1), the GenBank accession number and coordinates relative to the translation start site are listed. Note: G nucleotides at position 14 have been shaded in grey to illustrate a hint of systematic gene-specific differentiation of the motif that can be detected between MTT5 and the MTT1-MTT3 T. thermophila gene pair. (0.07 MB DOC) [file pone.0000291.s003.doc]

**Table S3. MTCM1 motif copies found in the entire *T. thermophila* genome and upstream of the TpMT-1 gene: Sequences, match locations, and nearest gene**

| Match in canonical orientation | Scaffold | Scaffold location | Nearest gene | Gene annotation |
| --- | --- | --- | --- | --- |
| ATGTGAATCATTAAT | 8254373 | 445472 | *MTT1* | Metallothionein |
| TTGTGATTCTTGAAT | 8254373 | 445568 | *MTT1* | Metallothionein |
| GATTGACTCATGATT | 8254373 | 445637 | *MTT1* | Metallothionein |
| GATTGACTCATGATT | 8254373 | 445654 | *MTT1* | Metallothionein |
| ATGTGATTCTTGAAT | 8254373 | 445706 | *MTT1* | Metallothionein |
| ACGTGATTCACGATT | 8254373 | 445771 | *MTT1* | Metallothionein |
|  |  |  |  |  |
| ATGTGATTCTTGAAT | 8254373 | 447793 | *MTT3* | Metallothionein |
| GACTAAATCAAGAGT | 8254373 | 447875 | *MTT3* | Metallothionein |
|  |  |  |  |  |
| GTGTGATTCTTGAAT | 8254577 | 37193 | *MTT5* | Metallothionein |
| ATGTGATTCATGAGT | 8254577 | 37257 | *MTT5* | Metallothionein |
| ATGTGAATCATGAGT | 8254577 | 37286 | *MTT5* | Metallothionein |
| GTGTGAATCATGAGT | 8254577 | 37392 | *MTT5* | Metallothionein |
| GAGTGAATCATGAGT | 8254577 | 37446 | *MTT5* | Metallothionein |
| GTGTGAATCATGAGG | 8254577 | 37498 | *MTT5* | Metallothionein |
| GTGTGAATCATGAGT | 8254577 | 37554 | *MTT5* | Metallothionein |
| ATGTGATTCATGAGT | 8254577 | 37703 | *MTT5* | Metallothionein |
| TAGTCACTCATGAAT | 8254577 | 37708 | *MTT5* | Metallothionein |
| GTGTGAATCATGAGT | 8254577 | 37809 | *MTT5* | Metallothionein |
| GAGTGAATCATGAGT | 8254577 | 37863 | *MTT5* | Metallothionein |
| GTGTGAATCATGAGG | 8254577 | 37912 | *MTT5* | Metallothionein |
| GTGTGAATCATGAGT | 8254577 | 37968 | *MTT5* | Metallothionein |
|  |  |  |  |  |
| ATGTGATTCTTGAAT | 8254379 | 304703 | 28.m00251 | Hypothetical |
| TTGTGATTCTTGAAT | 8254448 | 69882 | 165.m00075 | Rhodopsin-like |
| ATGTGAATCATTAAT | 8254487 | 83242 | 63.m00169 | Hypothetical |
| GATTGACTCATGATT | 8254819 | 1505496 | 2.m02410 | Cobalamin synthesis |
|  |  |  |  |  |
| ATGTGATTCTTGAGC | AJ005080 | -456 | *TpMT-1* | Metallothionein |
| AAGTGATTCTTGAGC | AJ005080 | -389 | *TpMT-1* | Metallothionein |
| TAGTGATTCCTGAAT | AJ005080 | -310 | *TpMT-1* | Metallothionein |
| AAGTGATTCCTGAGT | AJ005080 | -234 | *TpMT-1* | Metallothionein |
| AAGTGATTCTTGGAT | AJ005080 | -160 | *TpMT-1* | Metallothionein |

Matches: listed in the same orientation; TCA and TGA trinucleotides have been highlighted in aqua and yellow to facilitate motif sequence comparisons and identification of the AP-1-binding-related element (TGANTCA). Nearest genes: named MT genes or predicted gene model identifiers; sequence, coordinates, functional annotation and EST support for gene models are available by searching the Tetrahymena Genome Database (<http://www.ciliate.org/>). For the *T. pyriformis* MT-1 gene (*TpMT-1*), the GenBank accession number and coordinates relative to the translation start site are listed. Note: G nucleotides at position 14 have been shaded in grey to illustrate a hint of systematic gene-specific differentiation of the motif that can be detected between *MTT5* and the *MTT1-MTT3* *T. thermophila* gene pair.
