# Supplementary material for: Tetrahymena Metallothioneins Fall into Two Discrete Subfamilies
Source: PLoS One. 2007 Mar 14;2(3):e291. doi: 10.1371/journal.pone.0000291 (PMC1808422; doi:10.1371/journal.pone.0000291)
Supplement: Table S4 — Quantitative real-time RT-PCR standard curve parameters for each MT gene and the expression control (0.03 MB DOC) [file pone.0000291.s004.doc]

**Table S4**. **Quantitative real-time RT-PCR standard curve parameters for each MT gene and the expression control**

|  | α - tubulin | MTT1 | MTT3 | MTT5 |
| --- | --- | --- | --- | --- |
| Slope (S) | - 3.22 | - 3.18 | - 3.27 | - 3.34 |
| PCR efficiency (%) | 99.9 | 99.6 | 99.4 | 99.1 |
| R^2 a^ | 0.99 | 0.99 | 0.99 | 1 |

^a^ correlation coefficient.
