# Supplementary figures and images for: Tetrahymena Metallothioneins Fall into Two Discrete Subfamilies
Source: PLoS One. 2007 Mar 14;2(3):e291. doi: 10.1371/journal.pone.0000291 (PMC1808422; doi:10.1371/journal.pone.0000291)

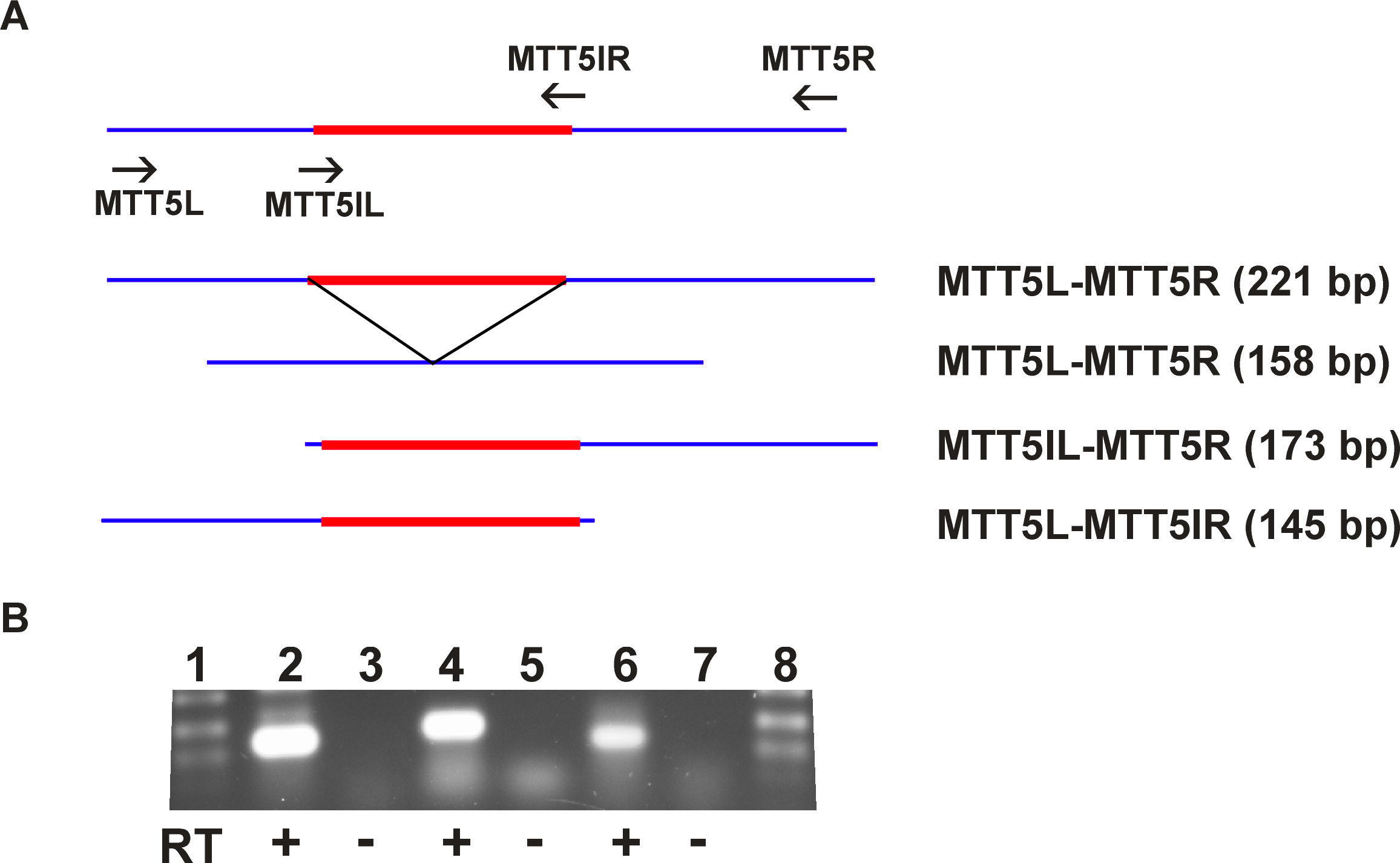

Supplement: Figure S1 — Alternative Splicing of the MTT5 3′UTR intron. A. Location of primers used for RT-PCR relative to the intron in the 3′ UTR of MTT5. The intron is shown in red, while the flanking regions are blue. The RT-PCR products produced by each set of primers (arrows) and their expected sizes are shown below. Primer sequences are shown in Table S1. B. RT PCR analysis using RNA extracted from conjugating Tetrahymena cells. Lanes 1 and 8: 1 kb DNA ladder (Invitrogen). Lanes 2 and 3: PCR products produced by primers flanking the intron (MTT5L and MTT5R). The predicted 158 bp product (templated by spliced mRNA) and a fainter 221 bp product (templated by unspliced RNA) are RT-dependent, i.e., seen only in the “+RT” (plus reverse transcriptase) lane. Lanes 4 and 5: one primer spans the left boundary of the intron (MTT5IL) and the other flanks it on the right (MTT5R). The PCR product is RT-dependent and has the size (173 bp) expected if it is templated by unspliced RNA. Lanes 6 and 7: the left flanking primer (MTT5L) in combination with a primer which spans the right hand junction of the intron (MTT5IR). The PCR product is RT-dependent and has the size (145 bp) expected if it is templated by unspliced RNA. (0.35 MB TIF) [file pone.0000291.s005.tif]
